# Integrated analysis of cellulose structure and properties using solid-state low-field H-NMR and photoacoustic spectroscopy

**DOI:** 10.1038/s41598-024-80069-y

**Published:** 2025-01-16

**Authors:** Levente Csóka, Worakan Csoka, Ella Tirronen, Ekaterina Nikolskaya, Yrjö Hiltunen, Bunsho Ohtani

**Affiliations:** 1 Faculty of Informatics, ELTE Eötvös Loránd University, Budapest, 1053 Hungary; 2 Institute of Cellulose and Paper Technology, Celltech-Paper Ltd, Lócs, 9634 Hungary; 3 FiberLaboratory, South-Eastern Finland University of Applied Sciences, 57200 Savonlinna, Finland; 4 Nonprofitable Organization Touche NPO, Sapporo, 060-004 Japan

**Keywords:** Cotton cellulose fibres, Solid-state low-field proton NMR, Crystallinity, Reversed double-beam photoacoustic spectroscopy, Electron trap, Non-radiative recombination, Techniques and instrumentation, Materials science

## Abstract

In this study, we explore the structural intricacies of cellulose, a polymer composed of glucose monomers arranged in a linear chain, primarily investigated through solid-state NMR techniques. Specifically, we employ low-field proton nuclear magnetic resonance (H-NMR) to delve into the diverse hydrogen atom types within the cellulose molecule. The low-field H-NMR technique allows us to discern these hydrogen atoms based on their distinct chemical shifts, providing valuable insights into the various functional groups present in cellulose. Our focus extends to the examination of anomeric protons of glucose units and protons linked to carbon atoms engaged in glycosidic linkages within cellulose chains, which exist in diverse crystalline and amorphous forms. Solid-state low-field H-NMR spectroscopy aids in characterizing the crystallinity degrees and amorphous regions within cellulose, revealing time-dependent changes in free induction decay (FID) signals. Complementing this, we investigate the photo-absorption properties of cellulose fibers under both continuous and modulated irradiation using reversed double-beam photoacoustic spectroscopy (RDB-PAS). This photoacoustic approach allows us to observe ultraviolet- and visible light-induced processes, including electron trap filling and reductive changes on the fiber surface. Our findings suggest that RDB-PAS is a feasible method for estimating the electron trap distribution, serving as a potential measure of the density of crystalline cellulose defects. This integrated approach of combining solid-state low-field H-NMR and RDB-PAS techniques offers a comprehensive understanding of cellulose structure and properties, enhancing our ability to characterize its diverse features.

## Introduction

Cellulose, a pivotal carbohydrate in structural and functional biology, plays a fundamental role in cellular and tissue architecture. Its significance emanates from two primary sources: the outcome of photosynthetic processes in plants and the intricate bio-processing orchestrated by bacteria. Notably, cellulose synthesis was harnessed through the ingenious cellobiosyl fluoride pathway, yielding intricate spherulite structures^1^.

Cellulose consists of glucose monomers linked together in a linear chain, which were extensively studied using solid-state NMR techniques over several decades^2–4^. Initial research pioneered by early studies established critical insights into cellulose structure through carbon-13 NMR, while more recent advancements, including dynamic nuclear polarization (DNP) techniques, have provided unprecedented detail on molecular architecture^5–10^. These studies, typically conducted at high magnetic fields using high-resolution carbon-13-based multidimensional solid-state NMR, enable precise differentiation of molecular environments in cellulose through chemical shifts and connectivity patterns, facilitating the identification of functional groups and structural elucidation with exceptional detail. However, in our study, we utilize low-field proton NMR. This approach is particularly advantageous for examining the free induction decay (FID) signals, offering enhanced sensitivity to the dynamics of hydrogen atoms in both crystalline and amorphous regions of cellulose. This complements high-field, high-resolution carbon-13-based multidimensional solid-state NMR, which excels in providing detailed structural insights into cellulose. By using low-field proton NMR, however, we achieve enhanced sensitivity to hydrogen dynamics, allowing us to effectively monitor time-dependent changes and distinguish between crystalline and amorphous regions. These aspects provide valuable insights into the cellulose structure, particularly in contexts where sensitivity to hydrogen mobility is critical.

The distinct attributes of cellulose particles define their widespread utility, characterized by low density, mechanical robustness^11,12^, resistance to various chemical environments, and a unique combination of hydrophilicity and thermal stability^13^. At the molecular level, cellulose particles exhibit fascinating properties, including a notable surface zeta-potential averaging − 40 mV^14^, attributed to the abundance of hydroxyl groups. This isoelectric point, found within the pH range of 2 to 3, underlines cellulose’s intricate interaction with its surroundings^15^.

These exceptional properties have propelled cellulose into diverse applications, from electromechanical^16^ and magnetic responses to cutting-edge display technologies^17^, sensors^18^ and innovative biomedical tools, solidifying its position as a material of boundless potential.

Photoacoustic spectroscopy (PAS) is a powerful technique for studying light-matter interaction, providing valuable insights into material properties. Reversed double-beam photoacoustic spectroscopy (RDB-PAS) enhances measurement capabilities by eliminating noise sources, improving sensitivity and accuracy. In this manuscript, we explore the intricacies of cellulose’s structure and properties using electromagnetic and magnetic fields. The study aims to investigate the ordered and less ordered molecular arrangement in cellulose fibres after chemical processes using solid-state low-field proton NMR and RDB-PAS.

## Materials and methods

### Materials

We sourced Whatman filter paper, crafted from cotton linters, through GE Healthcare. Additionally, we obtained polyvinyl alcohol (86.5–89% hydrolysed) and 30% hydrochloric acid from VWR Chemicals. All chemicals were used as received.

### Methods

The hydrolysis process: using HCl vapor occurred within a desiccator with a petri dish containing the HCl solution. To ensure complete air replacement with the HCl atmosphere inside the vessel, the desiccator valve remained open for 2 days. Subsequently, filter paper was introduced into the desiccator, and hydrolysis was allowed to progress for 2, 4, 6, and 8 h. Following hydrolysis, the filter paper underwent a 24-hour rinse in water to remove excess HCl. Finally, the filter paper was dried in a dryer at 60 °C.

Degree of polymerization (DP): it was determined by assessing the viscosity of 0.5% cellulose solutions, utilizing 0.5 M cupriethylenediamine (CED) as a solvent, and employing an automatic capillary viscometer following the T230 standard. For both untreated and hydrolyzed cellulose samples, a 25 ml water bottle containing copper rods was used. The samples were agitated until disintegration occurred. Subsequently, CED was introduced into the bottles, facilitating solubilization. The resulting solutions were then maintained in a constant temperature bath at 25 °C, and the viscosity of the solutions was measured using a capillary-type viscometer.

RDB-PAS measurement: followed a previously outlined protocol with specific modifications^19^. The photoacoustic spectroscopic measurement focused on absorbed photons generating acoustic waves. For this purpose, a homemade photoacoustic (PA) cell equipped with a MEMS microphone module and a quartz-glass window (transparent in the 250–1000 nm range) was utilized to minimize external influences. RDB-PAS measurements were conducted using wavelength-scanned monochromatic light (600 –250 nm) and a 35 Hz-modulated near-infrared (940 nm) LED. The RDB-PAS signal, representing the total trapped-electron density, was recorded without normalization. The experimental atmosphere for RDB-PAS was static methanol-saturated nitrogen. Prior to RDB-PAS, nitrogen gas saturated with methanol vapor flowed through the cell for 20–23 min at 30 ml/min.

The RDB-PAS measurement setup involved exchanging modulated- and continuous-light beams. In this process, continuous wavelength-scanning light excited electrons to enter electron traps (ETs), while simultaneously modulated monochromatic light detected the photoabsorption of electron-filled ETs, recording the photoinduced trap-filling spectrum. Before measurement, nitrogen gas saturated with methanol vapor flowed through the cell to irreversibly capture positive holes and prevent the disappearance of once-trapped electrons through reactions with positive holes.

Solid-state low-field 1H-NMR measurement: Untreated and acid-hydrolyzed cotton cellulose samples (Whatman filter paper) were meticulously chosen for the investigation of homonuclear dipolar interactions between H-H nuclei using solid-state low-field NMR. These samples underwent careful preparation, including drying at 105 °C for 72 h. They were then placed within borosilicate glass tubes measuring 0.8–1.0 mm in wall thickness, 10 mm in diameter, and 100 mm in length. Each tube was securely sealed with a rubber cap, ensuring optimal sample containment. After sealing, the tubes were placed in the low-field magnetic chamber and allowed to equilibrate for 30 s to align the spins along the low-field magnetic field before the radiofrequency spin relaxation started.

Our primary focus centred on the homonuclear interactions between hydrogen nuclei (H-H) within cellulose molecules, particularly at carbon 1–2 and 4–5 positions. These positions, characterized by the proximity of homonuclear nuclei, resulted in the generation of small magnetic moments. To explore these interactions, a low magnetic field of B_0_ = 0.5T was employed.

For the acquisition of ^1^H spectra, a direct polarization experiment was conducted. In this experiment, ^1^H nuclei were subjected to a short-duration (microseconds) radiofrequency pulse. The subsequent free induction decay (FID) was captured in the time domain following a 45° pulse. The sample’s hydrogen atom spins were observed to have a characteristic spin rate of approximately 26 kHz, which reflects their inherent stability and is crucial for maintaining optimal signal-to-noise ratios. This observation underscores the importance of the inherent properties of the samples in ensuring reliable spectral measurements. The obtained FID curves underwent deconvolution to eliminate instrumental noise and enhance data quality. Subsequent analysis utilized PeakFit software, enabling the extraction of relevant information from the FID line shapes in both the time and frequency domains.

## Results

**Fig. 1:**
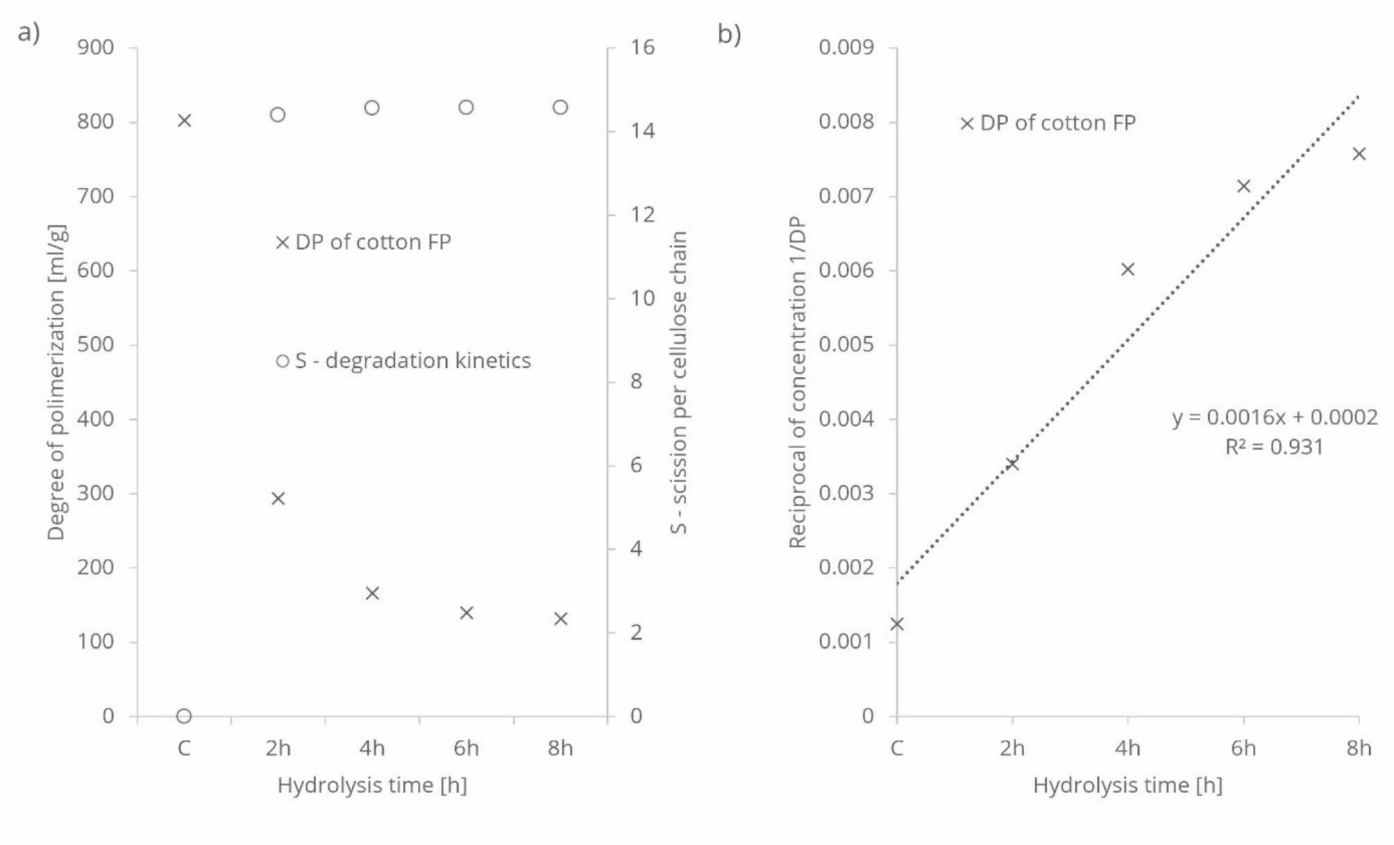
Reduction of the DP of cellulose as a function of time (**a**) and reaction kinetic fits (**b**).

HCl hydrolysis and the degree of polymerization of cotton filter paper were investigated, and the results are presented in Fig. 1a) and b). During HCl hydrolysis, vapor was adsorbed on the surface of cotton fibres, targeting the disordered segments (amorphous segments), resulting in a leveling-off degree of polymerization around 132 mgg⁻¹^20^. As hydrolysis progressed, the degradation of amorphous segments increased rapidly until reaching the crystalline segments. The degree of polymerization decreased rapidly, as shown in Fig. 1a), consistent with literature values^21^. The structural integrity of crystalline and amorphous parts was preserved, as indicated by the small amount of mass loss (data not shown). The original hierarchical structure of the fiber matrix still held the cellulose nanocrystals together after HCl vapor hydrolysis.

During cellulose degradation, both weak links and glycosidic bonds in the amorphous region were cleaved first. These two simultaneously occurring mechanisms can be described by the equation (which are plotted on Fig. 1a):

$$\:S={n}_{w}^{0}\left(1-{e}^{-{h}_{0}{k}_{w}t}\right)+{n}_{a}^{0}(1-{e}^{-{h}_{0}{k}_{a}t})$$

where $\:{n}_{w}^{0}=9.31$ and $\:{n}_{a}^{0}=5.24$ are the initial number of cellulose bonds (weak links and of glycosidic bonds in the amorphous regions) available for the degradation. $\:{k}_{w}$ and $\:{k}_{a}$ are the first order reaction kinetic rate constants and $\:t$ is time in min. Each cellulose bond degraded with its rate, making *S* the sum of parallel first-order processes—the number of scissions per cellulose chain. The solid/vapor degradation is analogous to a solid/liquid system^22^.

The HCl-initiated degradation of cotton cellulose FP occurred at a vapor pressure of 2.38 kPa, which increased collisions and, consequently, the kinetic energy of the chemical degradation rate. This process can be characterized by second-order type reaction kinetics. The second-order kinetic fit to the cotton cellulose FP degradation is illustrated in Fig. 1b).

### ^1^H-NMR characterisation

The diamagnetic^23^ nature of cellulose material implies the absence of a magnetic moment. When exposed to a low-field 0.5 T external, permanent magnetic field around solid-state cellulose samples, a magnetic moment is induced in the opposite direction of the main magnetic field.

Upon excitation by a radio frequency pulse, the induced magnetic moment leads to non-equilibrium nuclear spin magnetization precessing about the magnetic field. Within cellulose molecules, protons’ spin alignment in the T2 plane is induced by the radio frequency pulse, resulting in spin-spin relaxation and subsequent diphasic relaxation. The duration of dephasing corresponds to distinct signals in the free induction decay (FID) time domain, providing insights into variations in molecular interactions within crystalline and amorphous cellulose^24^.

**Fig. 2:**
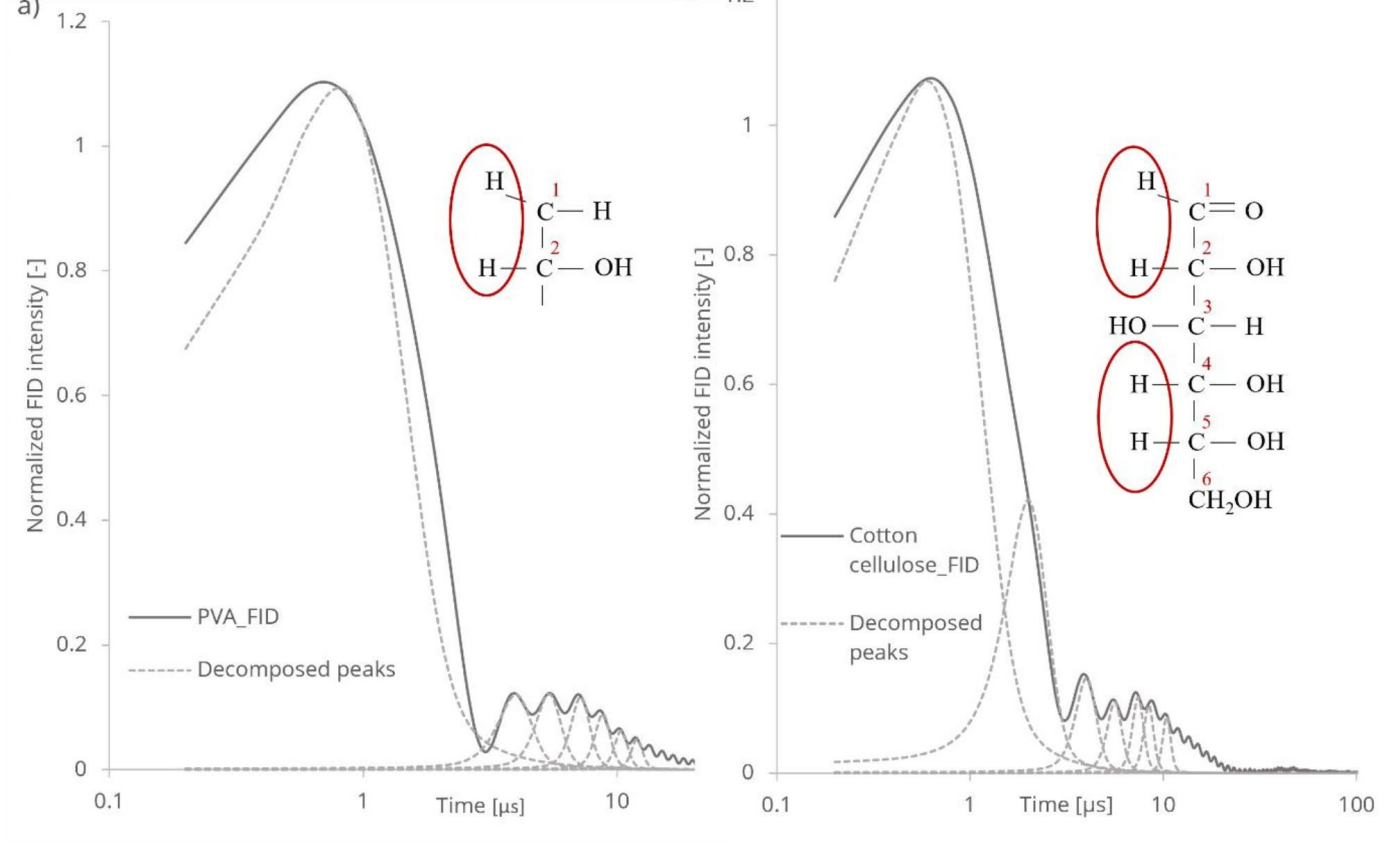
Free induction decay relaxation curves for (**a**) PVA and (**b**) cotton cellulose. The dashed lines are the deconvoluted spectra, and the chemical formulas indicate the proton locations, where the spin-spin relaxations were observed.

The envelope curve of this observable NMR signal, referred to as spin relaxation, exhibits characteristic feature related to cellulose material^25^. The spin relaxation process accompanies the free induction decay signal, which can be divided into two main parts in the time domain 0.1-3 µs and 3–30 µs for both PVA and cotton cellulose samples. The diamagnetic polarizability of cellulose allows the observation of homonuclear dipolar interactions during spin-spin relaxation (refer to Fig. 2).

Relaxation curves for free induction decay were recorded for PVA (Fig. 2a) and cotton cellulose (Fig. 2b), and their characteristic transverse time components were deconvoluted.

Figure 2 illustrates that the structurally simple carbohydrate polyvinyl alcohol (PVA) exhibits an FID relaxation curve quite like that of cotton cellulose. Despite their different molecular chain conformations, both FID relaxation signals are a composite of several decomposed peaks. In the analysis, Voigt-type peaks was utilized for the deconvolution process, which is particularly advantageous for accurately representing the spectral features associated with cellulose. The Voigt profile is a convolution of Lorentzian and Gaussian functions, allowing for the effective modelling of peaks that may exhibit both sharp and broad characteristics. This duality is relevant in cellulose studies, where the interplay between structural ordering and dynamic behaviour can result to complex spectral shapes. By employing Voigt-type peaks, the highest R^2 (0.96–0.98) value was achieved during the fitting process, indicating a robust correlation between the experimental data and fitted model.

In the semi-crystalline PVA sample^26^, slow-resonating protons exhibit fast decay with one main peak. In contrast, the cotton cellulose sample displays two peaks, centred at 0.6 and 2 µs, characteristic of highly ordered intracrystalline regions in the microfibrils. These peaks are distinctive features of the ordered cellulose-chain regions, where strong and complex inter- and intramolecular hydrogen-bond networks form.

Native cellulose (cellulose I) found in nature features two intrachain hydrogen bonds: one in the O3-H···O5 and the other in the O2-H···O6 sections. Additionally, there is one interchain hydrogen bond in the O6-H···O3 section, linking the layers laterally and depending on the hydroxymethyl conformation at the C-6 position^27^.

The faster resonating protons contribute to the slow FID signal decay in the less-ordered cellulose chain inter- and intramolecular hydrogen bonds. The last, wide spectral line around 80 µs in Fig. 2.b originates from residual, bound water^28^.

Table 1 presents the characteristic features of the two main parts of the deconvoluted signals. The double line division in Table 1 data clearly indicates the two main parts. Differences observed in the less-ordered cellulose chains show a lowered centre of dephasing, indicating HCl vapor hydrolysis-driven crystallization.

**Table 1:** Properties of the cotton cellulose FP decomposed FID signal.

	Control	2 h	4 h	6 h	8 h
Centre time [µs]	0.60	0.60	0.60	0.60	0.60
	2.00	2.00	2.00	2.00	2.00
	4.00	4.00	4.00	4.00	4.00
	5.60	5.20	5.20	5.20	5.20
	7.40	7.40	7.40	7.40	7.40
	8.40	8.40	8.40	8.40	8.40
	10.40	10.60	10.40	10.40	10.40
FWHM*	1.18378556	1.2144756	1.22218589	1.22902419	1.14442863
Amplitude	1.06814779	1.08257591	1.07151413	1.06670517	1.0760861
	0.42159671	0.50027998	0.48491517	0.53467955	0.5204115
	0.14662238	0.1878341	0.18923013	0.20569357	0.1416223
	0.10921451	0.13480548	0.14738309	0.15161962	0.0968379
	0.11713145	0.1781789	0.17489104	0.18356857	0.1147520
	0.10449084	0.14127063	0.15371515	0.16408129	0.1024009
	0.08654341	0.10518312	0.11360613	0.12056752	0.0767149
Integrated area	1.3133108	1.35713761	1.34971711	1.34933856	1.28942229
	0.6049182	0.73554035	0.71725987	0.79508074	0.7229889
	0.21536877	0.2828798	0.28674657	0.31339461	0.2012691
	0.16151894	0.2041676	0.22460666	0.23233083	0.13835747
	0.17394835	0.2713754	0.268036	0.28288766	0.16482108
	0.15541507	0.2155022	0.23595653	0.25326102	0.14729986
	0.1290028	0.1608415	0.17478263	0.1865203	0.11058507

*Full Width at Half Maximum.

Decomposition curve type: Gauss-Lorentzian.

h – hydrolysis hour.

**Fig. 3:**
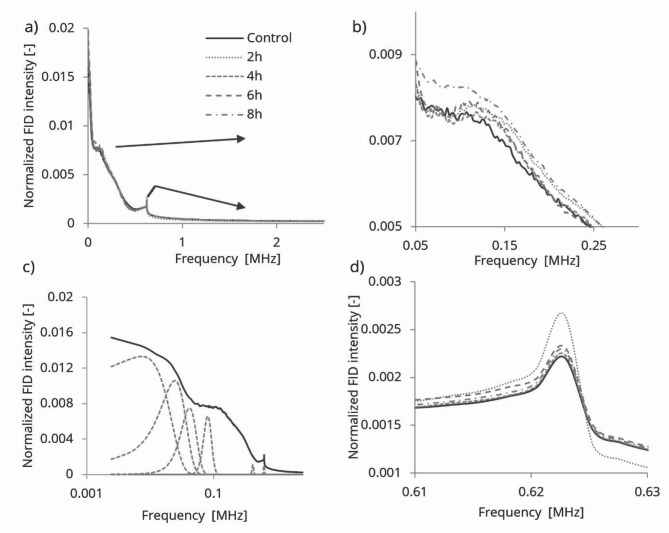
^1^H NMR Fourier spectra of the cotton cellulose FP. Full spectra (**a**), magnification of the arrow indicated regions (**b**) and (**d**), decomposed NMR Fourier spectra on log time scale.

Figure 3a-d present the NMR Fourier spectra of acid-hydrolyzed cotton cellulose samples. The spectrum in Fig. 3a exhibits a broad shoulder around 0.1 MHz and a sharp peak at 0.62 MHz. These two frequency locations are further enlarged in Fig. 3b and d. To better understand the spectral components, the NMR spectra were deconvoluted into harmonic Gauss-Lorentzian shape signals, as illustrated in Fig. 3c.

**Fig. 4:**
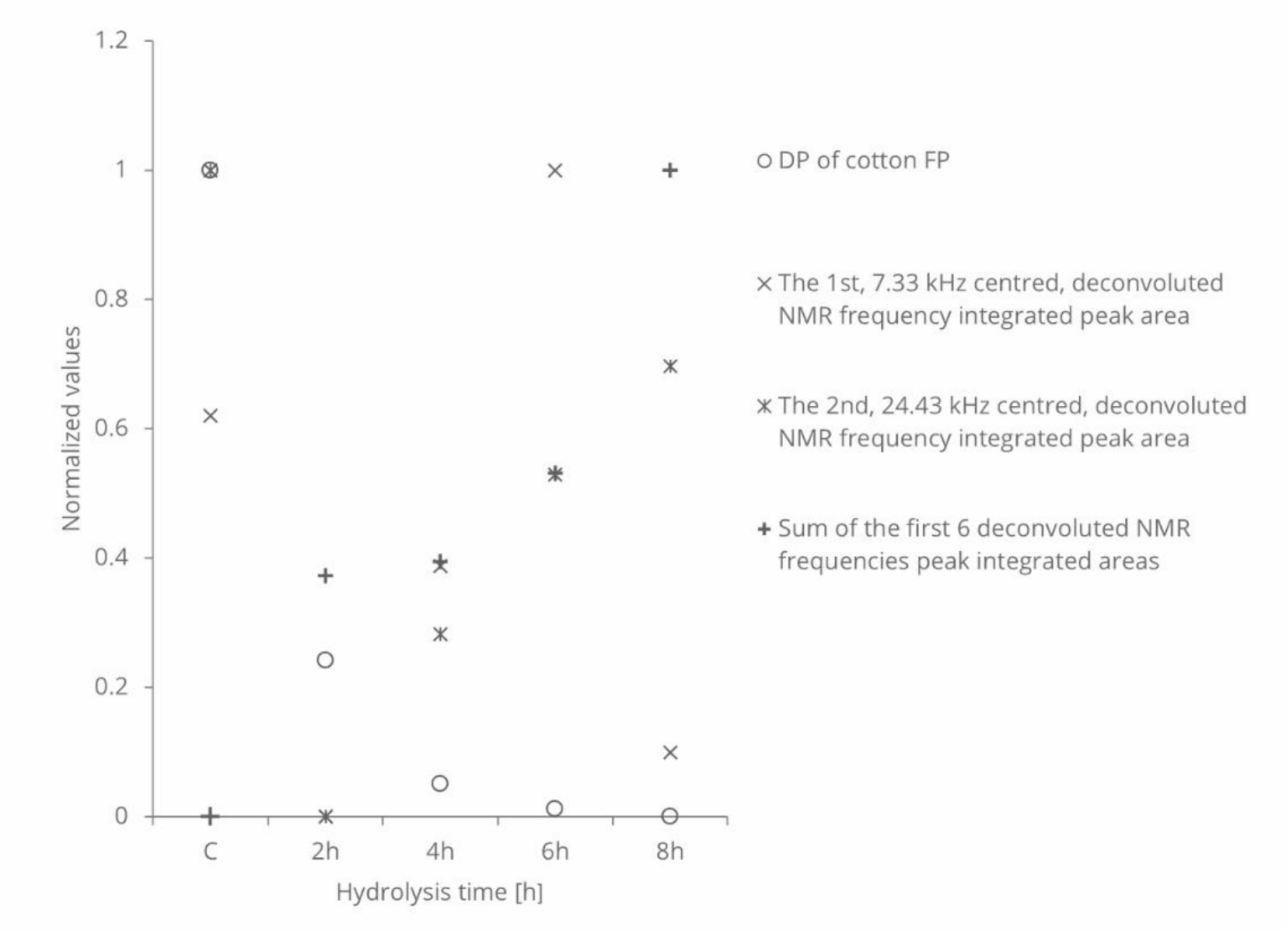
Normalized, deconvoluted ^1^H NMR Fourier spectra integrated areas of the cotton cellulose FP in comparison with normalized DP measurement.

The first and second decomposed NMR frequencies’ locations were found to be identical in both control and HCl vapor-treated samples, with a frequency ratio of 3.33. However, the integrated areas of these frequencies underwent changes. Figure 4 depicts the variations in the integrated areas, which are normalized and comparable with DP values.

The first frequency centred at 7.33 kHz and its integrated area initially decreased after 2 h of HCl vapor hydrolysis, gradually increased up to 6 h, and then reduced to almost the same level as the 2-hour treatment after 8 h of hydrolysis. In contrast, the second frequency centred at 24.43 kHz integrated area was maximum in the control sample, decreased to a minimum at 2 h, and then gradually increased up to 8 h. Summing up the integrated areas of the first 6 frequencies (up to 61.06 kHz), a continuous increase is observed. This incremental change in the integrated areas can be linked to a maximum 18% increase in crystallization. The ratio of crystalline to amorphous integrated areas indicates a 68.67% crystalline index, which increases by 7% after 8 h of hydrolysis. The calculation of the first increment value relies solely on frequencies related to fast decay, and some overlapping errors may account for the significant difference compared to the other increments. The crystalline index value is lower than the literature value^29^, but this difference could be attributed to the use of different measurement techniques.

Figure 4 unmistakably illustrates the occurrence of crystallization during HCl vapor hydrolysis, as discerned through low-field proton NMR measurement. This observation is substantiated by relative thermodynamic values supporting the hypothesis that crystallization predominantly takes place in the vapor phase rather than in an aqueous environment^20^. The higher heat of crystallization in the hydrated case, compared to the non-hydrated case, indicates a preference for crystallization under vapor phase conditions^30–34^.

Following the radio frequency excitation, precessing coupled spins interact through their dipolar coupling, resulting in split peaks in the frequency domain. The splitting pattern and the distance between the split peaks offer insights into the J-coupling constant. This constant quantifies the interaction between the coupled nuclear spins in the cellulose molecule, and its calculation is determined by the following equation:

$$\:J=\left(\varDelta\:\nu\:\right)/\left(\gamma\:{B}_{0}\right)$$

where J is the coupling constant, $\:\varDelta\:\nu\:$ is the frequency difference between the split peaks, $\:\gamma\:$ is the gyromagnetic ratio of the nucleus (^1^H=42.58 MHz/T), and $\:{B}_{0}$ is the strength of the static magnetic field. The J-coupling constant, being sensitive to molecular conformation and dynamics, serves as a valuable metric, enabling the determination of distances between nuclei in a molecule. The measured J-coupling constant is 0.68, almost identical to the literature data^34^, and results from reducing ring proton-proton decoupling, can be found in the control and 8 h hydrolysed samples as well. Additionally, the J-coupling constant of 1.83, identical to literature data^34^, is attributed to non-reducing ring H1-H2 proton decoupling in the 8 h hydrolysed sample. This information contributes to the understanding of bond lengths or conformations, as changes in the coupling constant magnitude reflect alterations in distance in the control and hydrolysed samples.

RDB-PAS measurement.

**Fig. 5:**
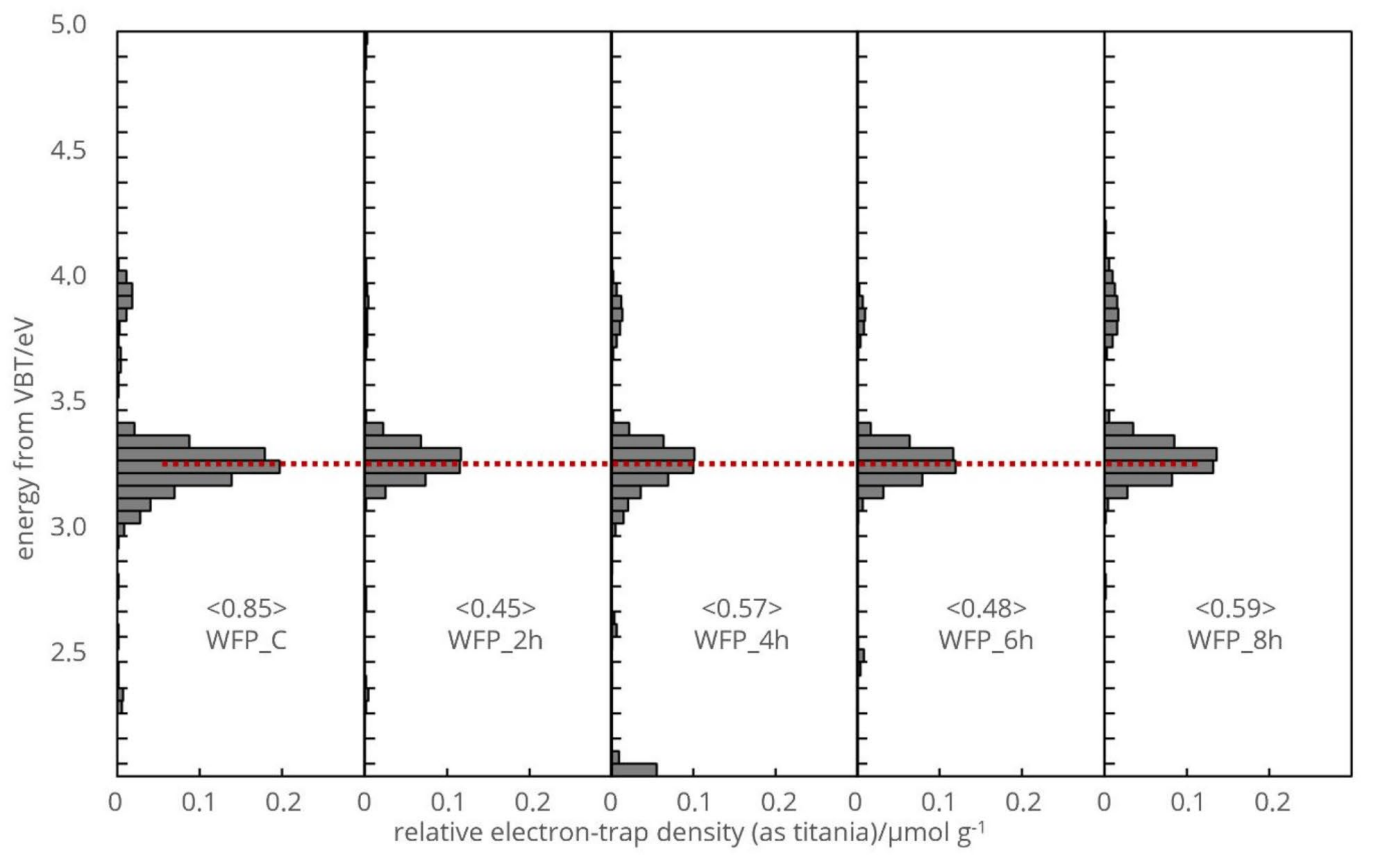
Energy-resolved distribution of electron traps in cotton cellulose FP samples under scanned, continuous light.

The energy distribution of electron traps (ETs) was integrated and subsequently differentiated from the lower energy side to unveil the energy-resolved distribution of ETs (ERDT) as a function of energy, as depicted in Fig. 5. Saturation of the RDB-PAS signal around 4.1 eV (300 nm) was consistently observed for both untreated and hydrolysed cotton cellulose FP samples using high-intensity LED. The total accumulated electron trap density was plotted against the photon energy of continuous light (E/eV = 1240/(wavelength/nm)). The radiation-less transition, competing or interfering with the radiative decay of electronically excited cellulose molecules, necessitated an independent photon field for its generation.

Under appropriate measurement conditions, all the ETs in these cellulose samples were filled with electrons upon UV irradiation, while there was a weak accumulation of electrons on the surface of the cellulose fibres during visible light irradiation.

Figure 5 presents the absolute ET density scale in $\:\mu\:mol{g}^{-1}{eV}^{-1};$ this scale, initially determined for titanium(IV) oxide (titania) samples, represents relative values for cellulose samples. ERDT plots in Fig. 5 exhibit significant results in the UV-visible irradiation zone, utilizing high-intensity 940 nm LED light.

Comparison of RDB-PAS results between the control and chemically treated cellulose samples revealed similar ERDT spectra. These patterns exhibited slight intensity variations between two main zones. The use of wavelength-scanned modulated light in the RDB-PAS study allowed for the measurement of spectra of electron-filled ETs with different energies, producing ERDT spectra indicating relative ET densities. Figures in brackets (< > ) in Fig. 5 denote total ET densities (relative value as titanium(IV) oxide). These values indicate that the crystallization did not initiate crystal defects to the same magnitude as observed in the control sample.

As reported previously^35^, total ET density is primarily dependent on the specific surface area and the main crystalline phase of samples, reflecting the size of the bulk (surface) and the bulk structure, respectively. The reduction in disordered regions by HCl vapor led to a decrease in ET densities, with subsequent crystallization increasing them. Differences in ERDTs are attributed to surface structure and are not reflected in total ET density.

## Summary

The effects of HCl vapor hydrolysis on the cotton cellulose surface were characterized through the analysis of free induction decay relaxation curves and the light absorption-induced interaction on crystal defects. Both proton NMR and RDB-PAS measurements, utilizing electromagnetic waves, were employed to comprehensively elucidate cellulose degradation and crystallization. Given the substantial proton linewidth in cellulose, the analysis primarily aimed to extract physical insights, such as crystallization, from the FID line shapes. While the broad proton linewidth posed limitations on discerning detailed chemical properties, the line shape analyses yielded rich physical insights.

Notably, HCl vapor hydrolysis of cotton FP did not introduce any surface charges, enhancing the efficiency of the H-NMR study, which focused on elucidating the clear surface properties of the fibres. By leveraging these techniques, our goal was to interpret homonuclear dipolar interactions within cellulose samples, contributing to a deeper understanding of this intricate molecular system.

## Data Availability

The datasets used and/or analyzed in the current study are available from the corresponding author upon reasonable request.
